# Continuing Treatment with *Salvia miltiorrhiza* Injection Attenuates Myocardial Fibrosis in Chronic Iron-Overloaded Mice

**DOI:** 10.1371/journal.pone.0124061

**Published:** 2015-04-07

**Authors:** Ying Zhang, Hao Wang, Lijing Cui, Yuanyuan Zhang, Yang Liu, Xi Chu, Zhenyi Liu, Jianping Zhang, Li Chu

**Affiliations:** 1 Department of Pharmacology, Hebei Medical University, Shijiazhuang, China; 2 Department of Chinese Materia Medica, Hebei Medical University, Shijiazhuang, China; 3 Department of Pharmaceutics, Hebei University of Chinese Medicine, Shijiazhuang, China; 4 The Fourth Hospital of Hebei Medical University, Shijiazhuang, China; Max-Delbrück Center for Molecular Medicine (MDC), GERMANY

## Abstract

Iron overload cardiomyopathy results from iron accumulation in the myocardium that is closely linked to iron-mediated myocardial fibrosis. Salvia miltiorrhiza (SM, also known as Danshen), a traditional Chinese medicinal herb, has been widely used for hundreds of years to treat cardiovascular diseases. Here, we investigated the effect and potential mechanism of SM on myocardial fibrosis induced by chronic iron overload (CIO) in mice. Kunming male mice (8 weeks old) were randomized to six groups of 10 animals each: control (CONT), CIO, low-dose SM (L-SM), high-dose SM (H-SM), verapamil (VRP) and deferoxamine (DFO) groups. Normal saline was injected in the CONT group. Mice in the other five groups were treated with iron dextran at 50 mg/kg per day intraperitoneally for 7 weeks, and those in the latter four groups also received corresponding daily treatments, including 3 g/kg or 6 g/kg of SM, 100 mg/kg of VRP, or 100 mg/kg of DFO. The iron deposition was estimated histologically using Prussian blue staining. Myocardial fibrosis was determined by Masson’s trichrome staining and hydroxyproline (Hyp) quantitative assay. Superoxide dismutase (SOD) activity, malondialdehyde (MDA) content and protein expression levels of type I collagen (COL I), type I collagen (COL III), transforming growth factor-β1 (TGF-β_1_) and matrix metalloproteinase-9 (MMP-9) were analyzed to investigate the mechanisms underlying the effects of SM against iron-overloaded fibrosis. Treatment of chronic iron-overloaded mice with SM dose-dependently reduced iron deposition levels, fibrotic area percentage, Hyp content, expression levels of COL I and COL III, as well as upregulated the expression of TGF- β_1_ and MMP-9 proteins in the heart. Moreover, SM treatment decreased MDA content and increased SOD activity. In conclusion, SM exerted activities against cardiac fibrosis induced by CIO, which may be attributed to its inhibition of iron deposition, as well as collagen metabolism and oxidative stress.

## Introduction

Iron is an essential element for cell metabolism and the function of various cellular enzymes, and its level is tightly regulated physiologically [1]. However, the body has no mechanism to excrete excess iron, which is highly toxic when present in high quantities and unbound from proteins [2]. Iron overload is a common clinical problem, arising from disorders of increased iron absorption, such as hereditary hemochromatosis or thalassaemia intermedia syndromes, or as a consequence of chronic blood transfusions for various blood disorders [3–5]. In these conditions, iron homeostasis is perturbed, and the excessive iron deposits in the liver, spleen, heart, bone marrow, pituitary, pancreas and central nervous system. Iron overload cardiomyopathy results from the accumulation of iron in the myocardium and is a leading cause of morbidity and mortality in patients with iron overload [6,7]. Iron overload cardiomyopathy, regardless of its origin, is characterized by a restrictive cardiomyopathy with early diastolic dysfunction which invariably progresses to a dilated cardiomyopathy [6]. Diastolic dysfunction occurs when the ventricle cannot fill properly due to an adverse accumulation and structural remodeling of the heart extracellular matrix (ECM) components defined as cardiac fibrosis [8–10]. The available body of evidence implicates iron itself in the initiation of fibrosis [11]. Additionally, in patients with iron overload the significant presence of myocardial fibrosis is a time-dependent process correlating with cardiovascular risk factors and cardiac complications [12].

For iron overload diseases, the current mainstays of therapy for excessive iron deposition in patients are phlebotomy and iron chelation, which are designed to remove whole-body iron [5,13]. Iron chelator was designed to bind with iron ions to remove the metal from the body. In accordance with its relatively high molecular weight and highly hydrophilic properties, chelators do not readily enter most types of cells (indirect action), including cardiomyocytes, but they may not prevent uptake of iron in organs, especially in those in which iron enters cells through specific ion channels [14,15]. Unfortunately, chelation therapy is cumbersome and associated with toxic adverse effects, including ophthalmological, auditory and bone toxicity and growth retardation [15]. For example, cardiac morbidity and mortality continue to occur in patients with thalassemia major treated with deferoxamine (DFO), a traditional iron chelator, presumably related to difficulties with adherence to chelation therapy [15,16]. However, phlebotomy and iron chelation are less effective for the treatment of myocardial fibrosis complicated with iron overload. Statin, baicalin and green tea were reported to inhibit or delay iron deposition *in vitro*, but their effects on iron-overloaded fibrosis have not been determined [17–19]. Encouragingly, recent studies have shown that calcium channels provide a major portal for iron uptake into cardiomyocytes in iron overload cardiomyopathy, and calcium channel blockers (CCBs) used in routine cardiovascular treatment can inhibit iron entry into cardiomyocytes and reduce the collagen volume in heart tissue [5,20–22]. Furthermore, a pilot trial investigating the effect of amlodipine (a CCB) on iron overload in patients with thalassemia major reported that it can serve as a complementary treatment to standard chelation regimens and may improve the efficacy of iron removal in the heart without the burden of significant side effects [23]. The above information suggests us that an ideal drug treatment should prevent iron accumulation and iron-related cardiac fibrosis, as well as have few side effects.

In China and other Asian countries, herbal medicines have been widely applied in clinical practice for more than two thousand years because they are easy to obtain, contain multiple components with efficacies against different ailments and generally display relatively low toxicity and side effects. *Salvia miltiorrhiza* (SM, also known as Danshen), a member of the *Labiatae* family, is a tonic herb of the nontoxic superior class used for improving microcirculation in traditional Chinese medicine. It has been used in Asian countries for multiple therapeutic effects in cardiovascular diseases including myocardial infarction, angina pectoris and atherosclerosis [24]. SM is highly valued for its dried roots or rhizomes, and its extracts contain several ingredients, including water-soluble phenolic acids (e.g., salvianolic acid B, danshensu) and lipophilic diterpene compounds (e.g., dihydrotanshinone and tanshinones) [25,26]. Accumulating studies have demonstrated that the protective effects of SM on the cardiovascular system may be attributed partly to its functional property as a CCB by reducing intracellular Ca^2+^ concentrations [24,27–30]. Moreover, the anti-fibrosis effect of SM is known to be triggered by various causes. Its potential mechanisms may involve reduced oxidant stress, diminished collagen synthesis in fibroblasts, inhibited transforming growth factor-β_1_ (TGF-β_1_) signal transduction and upregulated expression of metalloproteinases (MMPs) [24,31–35]. These studies suggest that SM may be beneficial in the treatment of cardiac iron-overload-induced fibrosis. Our previous studies also demonstrated the multi-targeted role of SM injection in decreasing iron deposition and inhibiting fibrotic development in iron-overloaded livers [36,37]. SM injection, an aqueous extract of SM, is a commercially available agent prepared according to the unified standard issued by the Ministry of Health of China and is commonly prescribed to treat cardiovascular, hepatic and renal diseases [38]. Based on high performance liquid chromatography with ultra violet (HPLC-UV) analysis in our previous studies, the three main constituents in SM injection include danshensu, protocatechuic aldehyde and salvianolic acid B [38,39], among which danshensu was shown to be a major marker for the antioxidant effect of SM water-extracts and could significantly inhibit β-adrenergic receptors-mediated cardiac fibrosis [40,41].

In the present study, we investigated the anti-fibrotic effects of SM injection in the heart by assessing a series of changes in histological and biochemical parameters in a mouse model established by chronic iron overload (CIO). We further explored the underlying mechanism of the effects of SM on cardiac fibrosis in this model by analyzing the key related oxidative stress markers and fibrosis-related molecules.

## Materials and Methods

### Drugs

SM Injection, prepared with the dried roots of the plant at the concentration of 1.5 g aqueous extract per milliliter (1.5 g/ml) and approved by the State Food and Drug Administration (approval no. Z32020161), was obtained from Shenlong Pharmaceutical Co., Ltd. (Jiangsu, China; batch no. 12050617). Desferrioxamine Mesilate (DFO) for injection was purchased from Novartis Pharma AG (Basel, Switzerland). Iron Dextran Injection was supplied by Sunaccord Biological Technical Co. Ltd. (Hunan, China). Verapamil Hydrochloride Injection was obtained from Harvest Pharmaceutical Co., Ltd. (Shanghai, China). Unless otherwise stated, other reagents were obtained from Sigma (Shanghai, China).

### Animals and Ethics Statement

Sixty male Kunming (KM) mice (22.0~25.0 g, 8 weeks old) were purchased from Hebei Medical University (certificate of conformity no. 1309104) and housed in rust-free cages at 20~22°C and 45~55% relative humidity on a 12-h light-dark cycle. All animal handling was carried out in accordance with the Guidelines of Animal Experiments from the Committee of Medical Ethics, Ministry of Health of China, and experiment procedures were approved by the Ethics Committee for Animal Experiments of Hebei Medical University (approval number: HEBMU-2013-09; approval date: September 04, 2013).

### Experimental protocols

Sixty KM mice were randomly divided into control (CONT), CIO, low-dose SM (L-SM), high-dose SM (H-SM), verapamil (VRP) and DFO groups (n = 10 in each group). Mice in the latter five groups were intraperitoneally (i.p.) injected with Iron Dextran Injection at 50 mg/kg once daily for 7 weeks. The first group served as the control and received isovolumic saline. Mice in the L-SM and H-SM groups were i.p. administered daily with 3 g/kg and 6 g/kg of SM Injection at 4 h before the administration of iron on the same day, while mice in the CONT and CIO groups received isovolumic saline. Mice in the VRP and DFO groups, as the positive control, were given i.p. Verapamil Hydrochloride Injection with 100 mg/kg and Desferrioxamine Mesilate with 100 mg/kg at 4 h before the administration of iron dextran on the same day. Food intake and activities of the mice were observed carefully every day, and the entire course lasted for 7 weeks. All evaluations were performed 24 h after the last iron administration.

At the end of the experiment, animals were anesthetized with sodium pentobarbital (50 mg/kg), and heart samples were removed and snap frozen in liquid nitrogen or fixed in 4% paraformaldehyde solution. None of mice used in our previous studies was reused in present study.

### Histological observations

The body weights and heart weights were collected. Myocardial tissues of all mice in each group were fixed in 4% paraformaldehyde solution for 24 h and embedded in paraffin. Using a microtome, 5 μm-thick serial sections were cut from the paraffin blocks and stained with hematoxylin-eosin (H&E) for routine histopathological observations according to the conventional procedure. The heart sections were processed with Prussian blue staining and Masson's trichrome staining for observation of iron deposition and myocardial fibrosis, respectively. A digital microscope with a multi-functional image analysis system (Leica DM 750 + DFC 450C, Leica Microsystems, Wetzlar, Germany) was used to analyze 20 randomly selected fields from each section at 400× magnification for determining the percent area of positive Masson's trichrome or Prussian blue staining, and representative micrographs of heart sections were taken for each group. The images were analyzed by following formula: % area = stained area / (parenchymal area—blank area) × 100.

### Hydroxyproline (Hyp) content

A colorimetric assay based on the reaction of oxidized Hyp with p-dimethylaminobenzaldehyde was used to measure Hyp content. A total of 50~100 mg of wet heart tissue was hydrolyzed for 20 min at 95°C in an acidic buffer solution. After the hydrolysis, the samples were centrifuged at 3500 rpm for 10 min. The absorbance of the final solution was evaluated by the colorimetric method at 550 nm, and Hyp content was calculated as μg per mg of tissue using a commercially available kit (Jiancheng Bioengineering Institute, Nanjing, China).

### Superoxide dismutase (SOD) activity and malondialdehyde (MDA) content

Excised hearts were rinsed in 1.15% KCl and homogenized in aqueous Tris-HCl buffer (100 mg tissue per milliliter of 50 mM phosphate buffer, pH 7.2). Homogenates were centrifuged at 10,000 × *g* for 20 min at 4°C to obtain the supernatant fractions used for analyses. The protein concentration of the supernatant was determined by the Lowry method using bovine serum albumin (BSA) as a standard. SOD activity and MDA content in heart tissues were detected by spectrophotometry using commercially available kits (Jiancheng, Nanjing, China).

### Immunohistochemistry

A manual procedure was performed according to instructions of the SP-9002 Histostain^TM^-Plus kit (ZYMED, CA, USA). Sections were deparaffinized, rehydrated and immersed in 10 mM citric acid (pH 6.0) to exclude epitope masking due to fixation. Sections were immunostained with primary antibodies against COL I (1:50 dilution, BA0325, Boster Bioengineering Co. Ltd., Wuhan, China) and COL III (1:50 dilution, BA0326, Boster Bioengineering Co. Ltd.) at 4°C overnight. Tissue sections were consecutively stained with streptavidin/peroxidase complex for 20 min at 37°C before a substrate solution of 3, 3′-diaminobenzidine tetrahydrochloride was added. Sections were then counterstained in hematoxylin. Bright field photographs were obtained with a digital camera connected to the microscope and analyzed with an image analysis system (Leica, Wetzlar, Germany).

### Western blot analysis

Total proteins were extracted from different tissues using a Tissue or Cell Total Protein Extraction Kit (Sangon, Shanghai, China) and normalized with a BCA Protein Assay Kit (Sangon, Shanghai, China). Frozen tissues (50 mg per sample) were homogenized in lysis buffer (Beyotime, Shanghai, China) and centrifuged at 14000 rpm for 10 min at 4°C. Denatured proteins (40 mg) were separated by sodium dodecyl sulfate-polyacrylamide gel electrophoresis (SDS-PAGE) and transferred onto a PVDF membrane. After blocking with 5% skim milk in Tris-buffered saline containing Tween-20, the membranes were incubated with rabbit anti-MMP-9 (1:500 dilution, 25C19, Beyotime), rabbit anti-TGF-β_1_ (1:500 dilution, 27C10, Beyotime) or rabbit anti-β-actin (1:500 dilution, AG019, Beyotime). Horseradish peroxidase-conjugated goat anti-mouse IgG (Zymed, San Francisco, CA, USA) was used as the secondary antibody. Hybridized bands were visualized using SuperSignal West Pico Chemiluminescent Substrate (Pierce, Rockford, IL, USA). The signals were quantified by densitometry. β-actin was used for as an internal protein control for normalization.

### Statistical analysis

Data were expressed as means ± standard error of the mean (SEM) and analyzed using the Statistical Package for Social Sciences (SPSS) for Windows version 15.0 software (SPSS Inc., Chicago, IL, USA). Differences among the groups were determined by one-way analysis of variance (ANOVA) followed by Student-Newman-Keuls multiple range tests. *P*<0.05 was considered significant.

## Results

### Effects of SM treatment on biometric parameters

During the experimental period, no mortality was observed in each group. Biometric parameters including body weight, heart weight and heart coefficients collected on the mice are presented in Table 1. The heart coefficients were calculated as the heart to body mass ratio × 100. The body weight gain in the CIO group was markedly lower than that in the CONT group (*P*<0.01). After treatment, the weight gain was significantly increased in the L-SM, H-SM, VRP and DFO groups compared with that in the CIO group (*P*<0.01 or *P*<0.05). The heart coefficient (heart weight to body weight ratio) in the CIO group was elevated by approximately 22.64% when compared that in the CONT group (*P*<0.01). By contrast, the heart coefficient declined by approximately 16.08% in the L-SM group and 18.47% in the H-SM group as compared to the CIO group (*P*<0.01), indicating that the increased heart coefficient was dose-dependently reduced by SM treatment. The VRP and DFO groups also showed significantly lower heart coefficients as compared to the CIO group (*P*<0.01).

**Table 1:** Effects of SM on body weight, heart weight and heart coefficient.

Group	Body weight (g)	Heart weight (g)	Heart coefficient (%)
CONT	41.21 ± 1.32	0.23 ± 0.01	0.53 ± 0.03
CIO	36.18 ± 2.55^##^	0.24 ± 0.02	0.65 ± 0.02^##^
L-SM	39.09 ± 2.83*	0.22 ± 0.01	0.56 ± 0.03**
H-SM	40.83 ± 3.02**	0.20 ± 0.02	0.53 ± 0.04**
VRP	38.95 ± 2.66*	0.21 ± 0.01	0.54 ± 0.02**
DFO	37.96 ± 3.29*	0.21 ± 0.02	0.55 ± 0.03**

Values are means ± SEM.

^##^ *P*<0.01 vs. CONT group;

*P<0.05,

**P<0.01 vs. CIO group.

### Effects of SM treatment on histological changes and iron deposition

**Fig 1:**
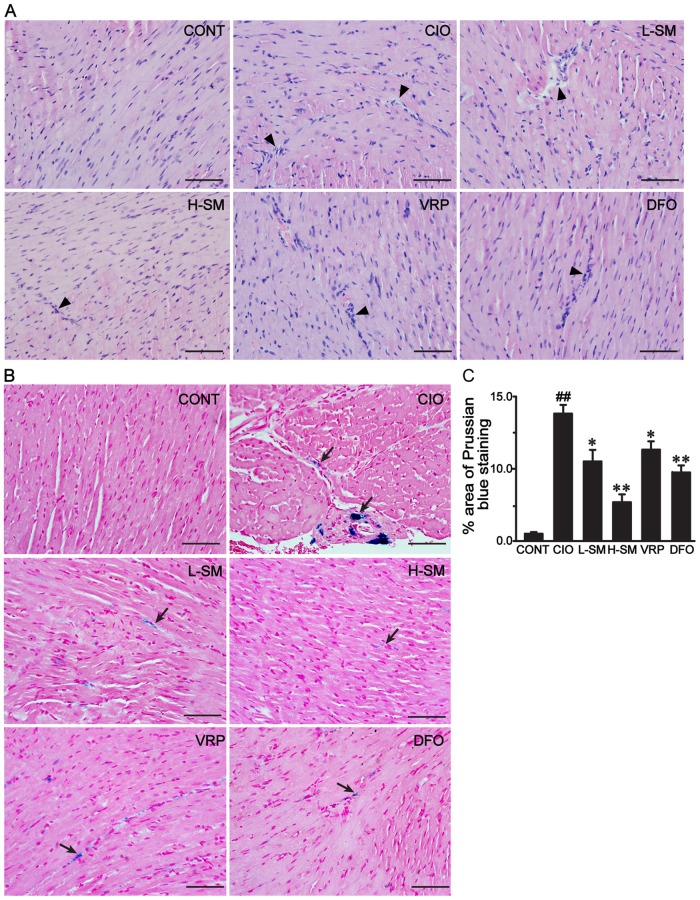
Effects of SM treatment on histological changes and iron deposition in mouse heart. Representative micrographs of H&E-stained heart tissues were obtained from each group to observe histological changes (A). The morphological location (B) and the percent area of iron deposition stained with Prussian blue (C) in each group are shown. The bar represents 50 μm (original magnification, 400×). Cardiac injury (triangles) and iron deposition (arrows) are indicated in these microscopic photographs, respectively. Results are means ± SEM. ##P<0.01 vs. CONT group; *P<0.05, **P<0.01 vs. CIO group.

As shown in Fig 1A, 1H and 1E staining was used for histological observation. Photomicrographs of heart tissue sections in the CIO group showed extensive myocardial atrophy, partial myocardial necrosis and fibrous tissue proliferation. After treatment with L-SM or H-dose SM, levels of pathological changes were markedly alleviated, as demonstrated by the regularly arranged cardiomyocytes and decreased fibrous tissue. Heart tissues in the VRP and DFO groups were restored to some extent.

Prussian blue staining was applied to visualize the iron deposition in heart tissue as shown in Fig 1B and 1C. Little staining was observed in hearts of the CONT group. Meanwhile, the CIO group displayed distinct iron deposition (dark blue) in heart tissue (Fig 1B), and the percent area of Prussian blue staining was noticeably elevated by approximately 13-fold compared to the CONT group (*P*<0.01). By contrast, SM treatment reduced the percent area of positively-stained tissue by approximately 38.64% (*P*<0.05) at the low dose and 65.91% (*P*<0.01) at the high dose, respectively, when compared with the CIO group. The percent area of Prussian blue staining in the VRP or DFO group also displayed a marked reduction as compared to the CIO group (*P*<0.05 or *P*<0.01).

### Effects of SM treatment on myocardial fibrosis

**Fig 2:**
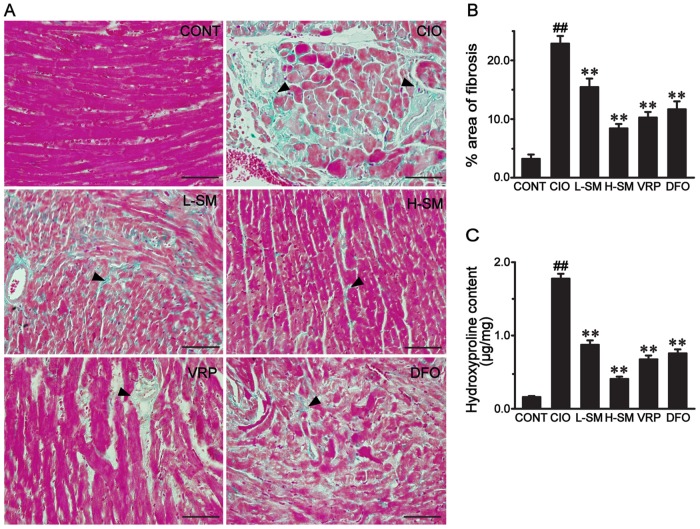
Effects of SM treatment on myocardial fibrosis. Representative micrographs were obtained from each group. The morphological location (A) and the percent area of myocardial fibrosis stained with Masson's trichrome staining (B) in each group are shown. The bright blue color represents the distribution of collagens (triangles), and the bar represents 50 μm (original magnification, 400×). The myocardial Hyp content was calculated in each group (C). Values are means ± SEM. ^##^ *P*<0.01 vs. CONT group; **P<0.01 vs. CIO group.

To detect the distribution of ECM components, changes in collagens indicative of fibrosis development were observed morphologically with Masson’s trichrome staining in heart tissue. As shown in Fig 2A, heart tissue in the CIO group presented a massive and intensive collagen accumulation (bright blue) in the disrupted myocardium. Compared with that in the CONT group, the percent area of fibrotic heart tissue in the CIO group significantly increased, reaching approximately 492.32% (Fig 2B, *P*<0.01). Upon administration with SM, areas of fibrotic heart tissue were dose-dependently diminished by approximately 30.08% in the L-SM group and 64.37% in the H-SM group, as compared with the CIO group (Fig 2A and 2B, *P*<0.01). Meanwhile, fibrotic cardiac tissue areas in the DFO and VRP groups were obviously lower than that in the ICO group (Fig 2A and 2B, *P*<0.01).

Hyp is unique to collagen and is a well-recognized quantitative marker for fibrosis. As shown in Fig 2C, the Hyp content (1.17 ± 0.28 μg/mg) of the CIO group was significantly higher than that of the CONT group (0.26 ± 0.11 μg/mg, *P*<0.01). After treatment with SM, the Hyp content decreased to as low as 0.89 ± 0.19 μg/mg in the L-SM group and 0.41 ± 0.20 μg/mg in the H-SM group (*P*<0.01, vs. CIO group). The Hyp contents in the VRP and DFO groups were 0.71 ± 0.15 μg/mg and 0.79 ± 0.17 μg/mg, respectively, lower than that in the CIO group (*P*<0.01).

### Effects of SM treatment on oxidative stress markers

**Fig 3:**
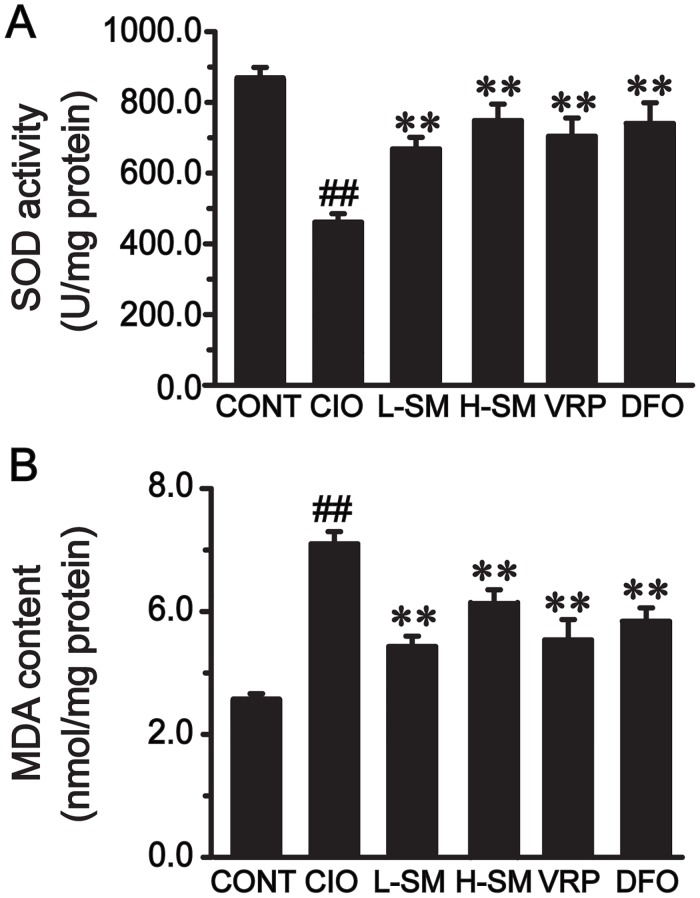
Effects of SM treatment on SOD activity and MDA content in heart homogenates. SOD activity (A) and MDA content (B) in each group are shown. Values are means ± SEM. ^##^ *P*<0.01 vs. CONT group; **P<0.01 vs. CIO group.

To assess the level of oxidative stress mediated by iron, two oxidative stress markers, SOD and MDA, were measured with heart tissue homogenates. Changes of SOD activity and MDA concentration in response to iron overload and treatment with SM are shown in Fig 3. Compared with that in the CONT group, SOD activity was notably reduced (CONT, 860.77 ± 16.16; CIO, 460.59 ± 17.53), while the MDA concentration was remarkably increased in the CIO group (*P*<0.01). Treatment with SM clearly elevated the SOD activity (L-SM, 671.12 ± 29.79; H-SM, 750.44 ± 35.04) and lowered MDA concentration in a dose-dependent manner, when compared with the CIO group (*P*<0.01). Significant alterations of SOD activity (VRP, 699.53 ± 31.00; DFO, 727.61 ± 44.71) and MDA concentration in the VRP and DFO groups were also detected when compared to the CIO group (*P*<0.01).

### Effects of SM treatment on expression of COL I and COL III assessed by immunohistochemistry

**Fig 4:**
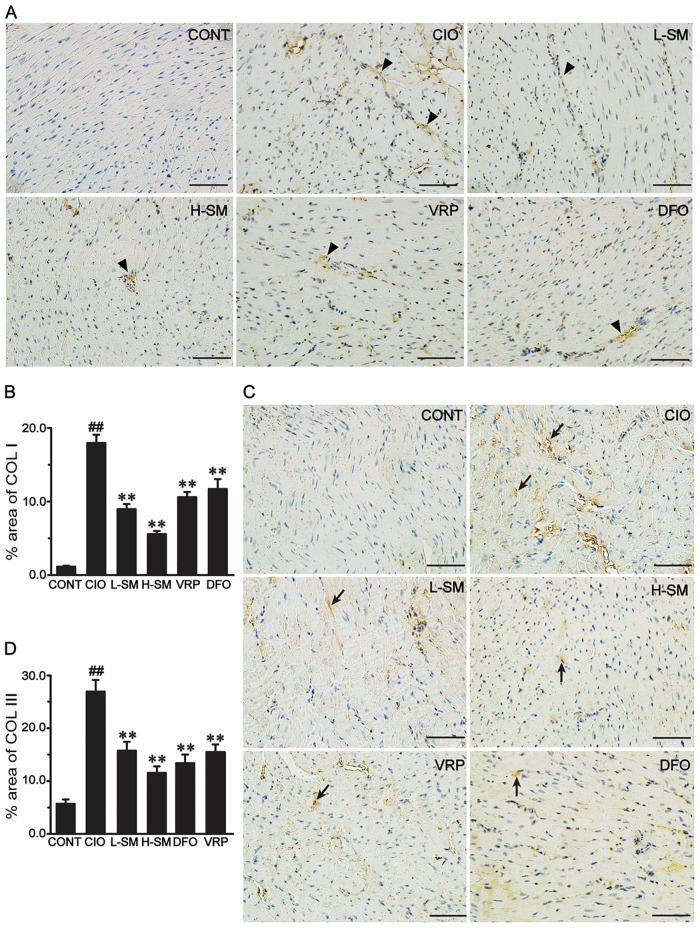
Effects of SM treatment on expression levels of COL I and COL III detected by immunohistochemistry. Representative microscopic photographs were obtained from each group. The morphological location and percent area of COL I expression (A and B, respectively) or of COL III expression (C and D, respectively) are shown. Positive expression of COL I (triangles) or COL III (arrows) is indicated, and the bar represents 50 μm (original magnification, 400×). Values are means ± SEM. ^##^ *P*<0.01 vs. CONT group; **P<0.01 vs. CIO group.

To further explore the underlying mechanisms of SM against heart fibrosis induced by iron, we detected the expression of COL I and COL III by immunohistochemistry, as shown in Fig 4. COL I was expressed at a low level in the CONT group. Compared with that in the CONT group, the percent area of COL I immunostaining significantly increased by approximately 10-fold in the CIO group (*P*<0.01). By contrast, SM treatment at either the low dose or high dose markedly reduced the expression of COL I by approximately 48.35% or 63.87%, respectively, as compared to the CIO group (*P*<0.01). Moreover, the percent area of COL I immunostaining in the VRP and DFO groups showed obvious decreases when compared to the CIO group (*P*<0.01). The expression of COL III in various groups exhibited a similar tendency with that of COL I (Fig 4C and 4D), and the increased expression of COL III was remarkably rescued by SM treatment in a dose-dependent fashion.

### Effects of SM treatment on expression of TGF-β_1_ and MMP-9 detected by Western blot

**Fig 5:**
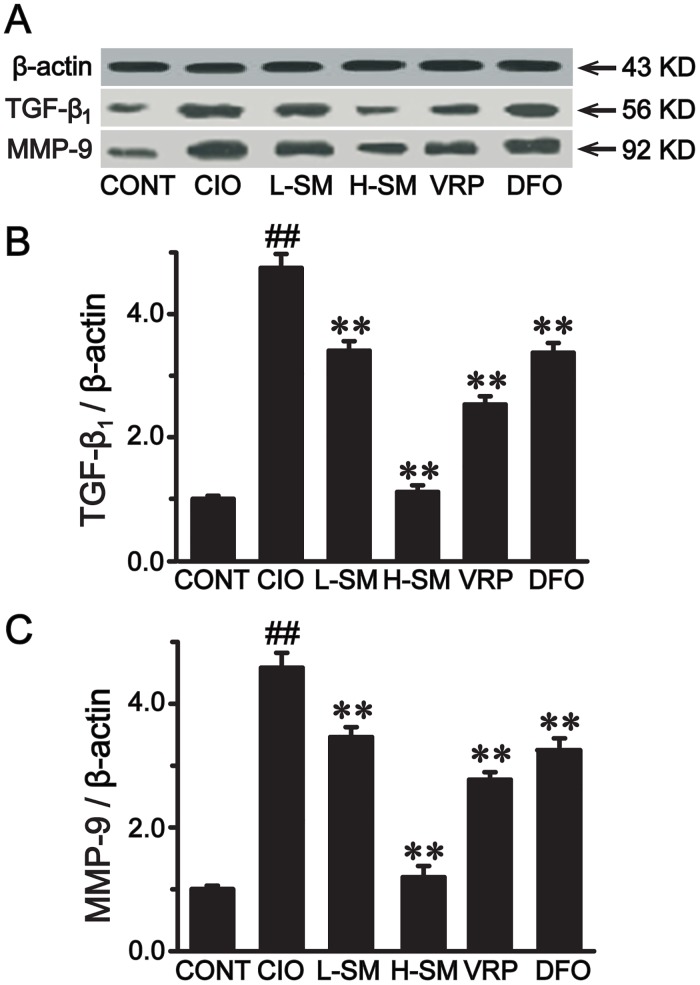
Effects of SM treatment on expression levels of TGF-β_1_ and MMP-9 by Western blot analysis. Representative immunoblots of TGF-β_1_ and MMP-9 expression in each group are shown (A). Relative intensities of TGF-β_1_ (B) and MMP-9 (C) were calculated by normalization to that of β-actin in each group. Values are means ± SEM. ^##^ *P*<0.01 vs. CONT group; **P<0.01 vs. CIO group.

TGF-β_1_, characterized by increased production of ECM components, strongly contributes to the pathogenesis of fibrotic disorders. As shown in Fig 5A and 5B, TGF-β_1_ expression was rapidly up-regulated by approximately 382.79% in the CIO group as compared to the CONT group (*P*<0.01). This increase of TGF-β_1_ expression was reduced by SM treatment at both the low dose and high dose by approximately 31.33% and 73.02%, respectively, when compared with the CIO group (*P*<0.01). Expression levels of TGF-β_1_ in the VRP and DFO groups noticeably declined compared with that in the CIO group (*P*<0.01).

MMP-9 (gelatinase B) is capable of denaturing and degrading ECM components. As shown in Fig 5A and 5C, MMP-9 expression in the CIO group was upregulated by approximately 367.81% compared with that of the CONT group (*P*<0.01). Meanwhile, this upregulation was rectified by SM treatment in a dose-dependent fashion. MMP-9 expression was reduced by approximately 25.11% in the L-SM group and 71.99% in the H-SM group, respectively, as compared to the CIO group (*P*<0.01). Moreover, the VRP and DFO groups also displayed low expression levels of MMP-9 when compared with that in the CIO group (*P*<0.01).

## Discussion

In the present study, we demonstrated the therapeutic potential of SM against cardiac fibrosis induced by CIO in a mouse model. Our data revealed that SM injection significantly reduced the heart coefficient, decreased iron deposition and ameliorated pathological changes in the heart of iron-overloaded mice. Furthermore, SM injection also markedly lowered the degree of fibrotic changes, decreased collagen synthesis and promoted collagen degradation, thereby inhibiting myocardial fibrosis induced by CIO. Thus, our study clarified the inhibitory effects of SM on iron-induced cardiac fibrosis.

In patients with both hereditary hemochromatosis and secondary hemochromatosis, iron is found predominantly within myocytes, leading to extensive myocardial fibrosis, disturbances of cardiac rhythm and even death [42]. Although iron chelation therapy is widely used to treat iron-overload conditions, recent studies have shown that iron overload cardiomyopathy is still the primary determinant of cardiac complications and survival in patients with iron overload [43,44]. For example, iron overload cardiomyopathy is responsible for more than half of the deaths in European, North American and Chinese patients with thalassemia major [3,45–47]. As a commonly used Chinese herbal medicine for cardiovascular diseases, SM has been demonstrated to have anti-fibrotic effects *in vitro*, *in vivo* and in clinical applications [48–51]. Additionally, our previous studies have demonstrated the significant protective effects of SM on the liver, heart and kidney in acute iron-induced injury [37–39]. Therefore, we hypothesized that SM treatment may be an effective therapy for myocardial fibrosis in chronic iron overload cardiomyopathy.

The model of secondary iron overload was established with i.p. injections of iron dextran at 50 mg/kg per day in mice. After seven weeks of iron overloading, the elevated organ coefficient (Table 1), abnormal histological changes (Fig 1A), increased areas of the heart with iron deposition (Fig 1B and 1C) accompanied by enlarged areas of fibrosis (Fig 2A and 2B) and high levels of Hyp (Fig 2C) were present, confirming the successful establishment of an iron-overload mouse model with iron imbalance and myocardial fibrosis. SM injection is widely accepted, and a pharmaceutical dosage form of SM is commercially available and readily acquired. Three main compounds of SM injection, danshensu, protocatechuic aldehyde and salvianolic acid B, were quantified using HPLC-UV in our previous studies [38,39]. Based on the formula for dose translation from one species to another and previous clinical studies on the best effects of SM injection, we selected 3 g/kg/day as the low dose and 6 g/kg/day as the high dose to investigate the inhibitory potency of SM on myocardial fibrosis induced by chronic iron overload [52–54]. The traditional iron chelator, DFO, and recently recognized iron blocker, CCB VRP, were used as positive controls to monitor the efficacy of SM treatment [5,55].

In the iron overload condition, the increased deposition of iron into the heart in the long term inevitably leads to chronic myocardial damage, which is commonly characterized by cardiomyocyte necrosis (disintegration), myocardial fibrosis, and consequently decreased body weight gain and increased the heart weight [6,56]. In our study, the capability of SM to remove iron in the iron-overloaded heart was demonstrated by the significantly decreased levels of iron deposits in the myocardium (Fig 2B and 2C) and restored myocardial architecture (Fig 2A), consistent with our previous studies [37,39]. More importantly, fibrotic cardiac tissue areas were also progressively diminished by administration of low and high doses of SM, while iron levels declined. Furthermore, a noticeable decrease of Hyp (Fig 3B), used as a marker in therapeutic trials for candidate anti-fibrotic drugs, was observed together with the profound decline in fibrotic tissue areas. These results suggest that SM could alleviate iron-mediated myocardial fibrosis in a dose-dependent manner. This finding was further supported by the observation of decreased areas of COL I and COL III expression in association with a dose increase of SM (Fig 4). Additionally, the increased body weight gain and reduced heart coefficient after SM treatment indirectly supported the efficacy of SM against iron overload-induced myocardial fibrosis (Table 1).

Although the exact mechanism of iron overload-induced heart fibrosis remains to be clarified, oxidative damage is believed to play a role in iron-mediated stimulation of cardiac fibroblasts, leading to increased myocardial fibrosis [12]. When iron levels are elevated, excessive free radical generation results in increased peroxidation and depleted antioxidants [43]. SOD is an endogenous enzymatic scavenger which can counterbalance the oxidative destruction of free radicals [57]. MDA, a representative product of lipid peroxidation, is highly toxic to cells [58]. In our study, fibrotic hearts of mice treated with SM showed an increase in antioxidant reserve, as displayed by the elevated SOD activity and decreased MDA content in both low-dose and high-dose SM groups (Fig 3). Previous experiments have demonstrated that oxidative stress acts on a profibrotic factor, and the increase of reactive oxygen species production drives promoter activity of COL I and stimulates collagen secretion [59,60]. Thus, we propose that the antioxidant activity of SM may be an essential mechanism involved in the anti-fibrotic effects of SM under iron overload conditions.

Although fibrosis is a multicomponent pathology driven by multiple factors, TGF-β_1_ is considered to be one of the major players in fibrosis development and has also been shown to decrease the expression and activity of MMPs which degrade the ECM (mainly collagens) [61]. Accumulating lines of evidence have shown that the binding of TGF-β_1_ to its receptors activates the Smad signaling pathway which induces the transcription of genes encoding components of the ECM [62,63]. To further explore whether profibrogenic molecules are associated with the anti-fibrotic effects of SM in the iron-overloaded heart, we examined expression levels of TGF-β_1_ and MMP-9 proteins. We found that the increase of TGF-β_1_ expression was restored by SM treatment at both low and high doses, together with the decline of elevated MMP-9 protein. Notably, high-dose SM reduced expression levels of TGF-β_1_ and MMP-9 by approximately 73.02% and 71.99%, respectively. These results implied that SM could effectively inhibit the fibrosis development in the iron-overloaded heart by suppressing ECM synthesis and promoting ECM degradation, which may be another potential mechanism for its inhibition of myocardial fibrosis induced by iron overload.

The present study also demonstrated effects of the positive controls, DFO and verapamil, on myocardial fibrosis mediated by iron. However, the ameliorating effects of DFO and verapamil were not superior to that of SM in the treatment of iron-overload-induced myocardial fibrosis. Moreover, DFO is associated with severe side effects, and its cumbersome administration schedule and parenteral infusions required on several days each week lead to poor compliance [15]. Although verapamil is well-documented to inhibit iron entry into cardiomyocytes, its anti-fibrotic effect in the iron-overloaded heart remains unknown. Combined our data with previous studies [35,48,64–74], although calcium channel blocker (verapamil) and iron chelator (DFO) offer mostly similar mechanistic effects as compared to SM, we tend to support that the efficacy of calcium channel blocker on iron-overloaded myocardial fibrosis is slightly better than that of iron chelator.

In conclusion, the present study showed that SM treatment effectively ameliorated myocardial fibrosis in chronic iron-overloaded mice. The potential mechanism of SM in decreasing fibrosis is likely attributed to the removal of excessive iron, inhibition of oxidative stress and regulation of ECM metabolism (synthesis and degradation).
